# Supplementary material for: Partial Characterization of Three Bacteriophages Isolated from Aquaculture Hatchery Water and Their Potential in the Biocontrol of Vibrio spp
Source: Microorganisms. 2024 Apr 29;12(5):895. doi: 10.3390/microorganisms12050895 (PMC11123731; doi:10.3390/microorganisms12050895)
Supplement: Supplementary file 1 [file microorganisms-12-00895-s001.zip › microorganisms-2966712-supplementary.pdf]

Supplementary Table S1. Homologues of *Vibrio* isolates according to the 16S ribosomal RNA gene

Isolates (this study)	Species and accession numbers	16S rDNA homology
Gdp1 (PP270122)	*Vibrio harveyi* (MT269608.1)	99.62%
Gdp3 (PP270123)	*Vibrio fluvialis* (CP053664.1)	99.91%
Gdp5 (PP270124)	*Vibrio fluvialis* (OY760100.1)	99.91%
Gdp6 (PP270125)	*Vibrio harveyi* (CP051123.1)	99.91%
Gdp8 (PP270126)	*Vibrio vulnificus* (MT052541.1)	99.54%
Gdp9 (PP270127)	*Vibrio chagasii* (MN938232.1)	99.37%
Gdp10 (PP270128)	*Vibrio sinaloensis* (MK995606.1)	99.79%
Gdp11 (PP270129)	*Vibrio chagasii* (LN832954.1)	99.36%
Gdp14 (PP270130)	*Vibrio harveyi* (KT982469.1)	98.74%
Gdp15 (PP270131)	*Vibrio crassostreae* (MT510175.1)	99.53%
Gdp16 (PP270132)	*Vibrio diabolicus (*OP565040.1)	99.19%
Gdp17 (PP270133)	*Vibrio harveyi* (PP053299.1)	99.90%
Gdp18 (PP270134)	*Vibrio alginolyticus* (MN938363.1)	99.04%
Gdp19 (PP270135)	*Vibrio anguillarum* (KC210822.1)	99.83%
Gdp20 (PP270136)	*Vibrio anguillarum* (CP022101.1)	99.83%
Gdp22 (PP270137)	*Vibrio harveyi* (KX904711.1)	99.83%
Gdp24 (PP270138)	*Vibrio alginolyticus* (MH643643.1)	99.23%
Gdp25 (PP270139)	*Vibrio harveyi* (MN938174.1)	99.25%
Gdp28 (PP270140)	*Vibrio harvey*i (MT071393.1)	99.54%
Gdp29 (PP270141)	*Vibrio harveyi* (MT605241.1)	99.35%
Gdp30 (PP270142)	*Vibrio anguillarum* (CP023310.1)	99.48%
Gdp31 (PP270143)	*Vibrio mediterranei* (MN945279.1)	96.96%
Gdp32 (PP270144)	*Vibrio azureus* (MH298563.1)	97.13%
Gdp33 (PP270145)	*Vibrio scophthalmi* (OM837045.1)	97.73%
Gdp34 (PP270146)	*Vibrio anguillarum* (LC365684.1)	98.00%
Gdp35 (PP270147)	*Vibrio scophthalmi* (OM837037.1)	98.38%
Gdp36 (PP270148)	*Vibrio qinghaiensis* (CP022741.1)	96.85%
Gdp37 (PP270149)	*Vibrio qinghaiensis* (CP022741.1)	98.43%
Gdp38 (PP270150)	*Vibrio scophthalmi* (OM837049.1)	98.42%
Gdp39 (PP270151)	*Vibrio scophthalmi* (OM837029.1)	98.59%
Gdp41 (PP270152)	*Vibrio ordalii* (KC884626.1)	98.43%
Gdp42 (PP270153)	*Vibrio neonatus* (MN939469.1)	96.96%
